# A Rare Case of a Traumatic Posterior Hip Dislocation in a 3-Year-Old Boy: A Case Report and Review of the Literature

**DOI:** 10.1155/2020/7560392

**Published:** 2020-03-09

**Authors:** Jan-Dierk Clausen, Marcel Winkelmann, Christian Macke, Philipp Mommsen, Christian Krettek, Stephan Brand

**Affiliations:** ^1^Trauma Department, Hannover Medical School, Hannover, Germany; ^2^Trauma Department, Klinikum Hochsauerland GmbH, Arnsberg, Germany

## Abstract

We present a rare case of neglected hip dislocation in a 3-year-old boy. Hip dislocations in childhood represent less than 6% of all injuries. The boy presented to the ED with ongoing hip pain after his leg got stuck in a carousel. The physical and radiologic examination revealed a posterior right hip dislocation. The closed reduction failed, so open reduction during surgery was performed. The postoperative protocol included 3 days of immobilization with early mobilization and pain-adapted weight bearing. No signs of femoral head malperfusion occurred 2 months after the injury. The patient did not complain of any limitations such as weight bearing problems or loss of range of motion. In comparison to adults, there are several specialties such as the fact that minor trauma can lead to hip dislocations due to the laxity of the ligaments, and due to the limited direct anamnestic options, neglected hip dislocations can occur. The treatment should focus on immediate proper reduction. The main complications after traumatic hip dislocation are avascular necrosis of the femoral head, redislocation, and early osteoarthritis.

## 1. Introduction

Hip dislocations in young children under 3 years of age are very rare. Overall, traumatic hip dislocations represent less than 6% of all traumatic injuries in children [1, 2]. Due to the special anatomical situation with laxity of the ligaments, even minor trauma can cause a dislocation of the hip in young children [3]. Just like in adult patients, posterior hip dislocations are much more common than anterior dislocations. A special group of patients is composed of children with neglected hip dislocations after minor trauma because the treating physician can be confronted with growth disturbances, muscular atrophy, and other complications. Nearly all neglected hip dislocations are posterior dislocations [4].

## 2. Case Scenario

**Figure 1:**
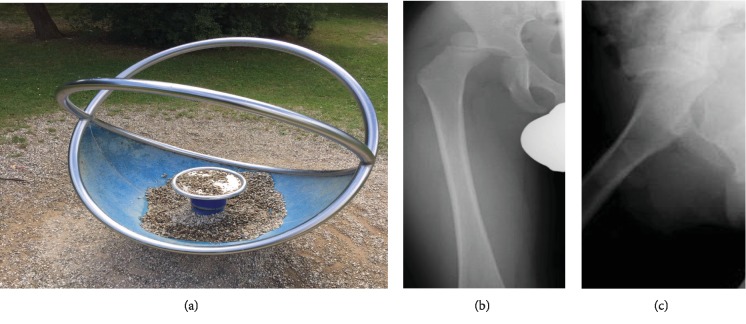
(a–c) Carousel (a); anteroposterior (b) and lateral (c) thigh X-rays showing the posterior luxated hip joint.

We present a case of a neglected traumatic posterior hip dislocation in a so far healthy and normally agile 3-year-old boy. He presented to our emergency department with severe pain in the region of the right hip joint. The mother reports that the pain started suddenly around 24 h after her boy played on a carousel two days ago (Figure 1(a)). While he was playing, the mother reported that her boy got stuck with his right leg in the carousel which kept on turning. So consecutively, the boy's leg got distorted.

**Figure 2:**
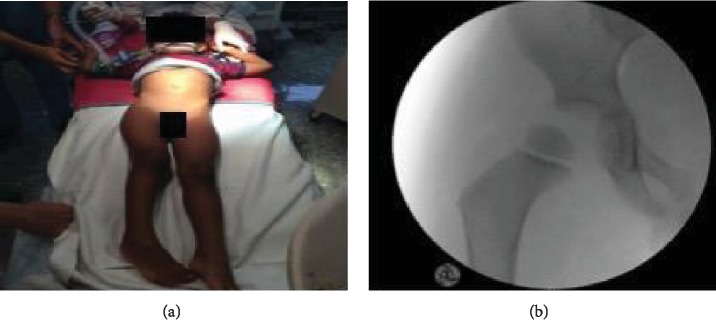
(a, b) Clinical findings before reposition and postinterventional image intensifier a.p. radiograph showing the incomplete reduction with persisting subluxation.

The clinical examination showed a 3-year-old boy in a normal nutrition state without any signs of maltreatment. His right lower extremity was compared to the left side shortened and an internal rotation imposed (Figure 2(a)). Any active or passive movement of the leg resulted in severe pain, and the patient refused to bear weight or even flex his hip joint. The perfusion and sensitivity of the leg were not affected, especially no signs for a lesion of the sciatic nerve revealed during the examination.

After clinical examination, anteroposterior and lateral hip X-rays were performed. They showed a posterior hip dislocation (Figures 1(b) and 1(c)).

Right after the diagnostic procedures, the patient was taken to a procedure room for closed reduction under general anaesthesia in standard technique. The postinterventional radiograph revealed a remaining subluxation of the left hip (Figure 2(b)). Therefore, a MRI scan including special perfusion sequences was performed immediately to assess the damage to the soft tissue and to assess the vascular situation of the femoral head directly after the injury.

**Figure 3:**
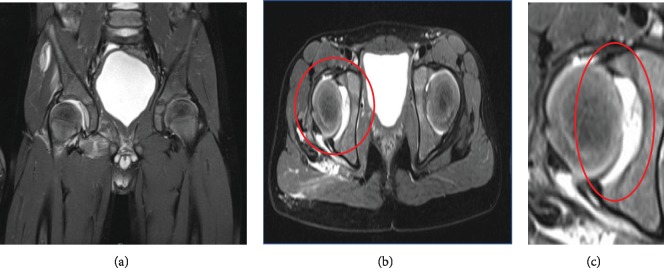
(a–c) Axial and coronal pd_tse_fs MRI pictures showing a type 1A lesion (Czerny classification) of the anterior-superior acetabular labrum and ruptured ligament of the femoral head.

The scan showed a type 1A lesion (Czerny classification) of the anterior-superior acetabular labrum (Figures 3(a)–3(c)). Additionally, it revealed a complete rupture of the ligament of the femoral head and an incomplete avulsion of the gluteus medius and external obturator tendon as well as intra-articular fluid (Figure 3(b)). The MRI suggests that the stumps of the ligament of the femoral head are preventing proper reduction. We found no initial signs for malperfusion of the femoral head.

**Figure 4:**
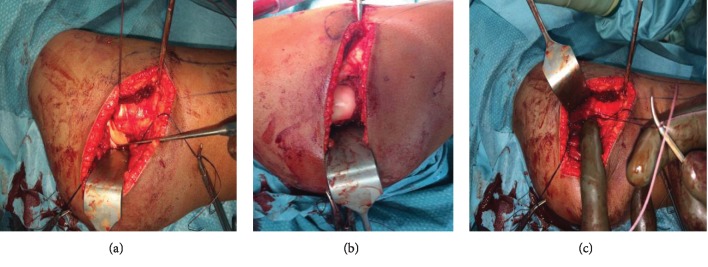
(a–c) Intraoperative findings showing the Kocher-Langenbeck approach with interposition of the inferior labrum (a), the intact cartilage of the femoral head (b), and the reconstructed capsule and muscles (c).

Due to the findings of the MRI scan, we urgently brought the patient to the operating theatre for open reduction and soft tissue management to prevent further damage to the femoral and acetabular cartilage and perfusion. The operation was performed in left lateral position using the Kocher-Langenbeck approach. The intraoperative findings confirmed the rupture of the femoral head ligament with a turned-up stump which makes a proper reduction impossible. Furthermore, the posterior inferior acetabular labrum was turned up into the joint. The cartilage surface was intact (Figures 4(a)–4(c)). After resection of the ligament and restoring the acetabular labrum, a proper reduction was possible and controlled by an intraoperative radiograph. Afterwards, the joint was clinically stable and the labrum was in anatomical position without any need of reconstructive sutures.

**Figure 5:**
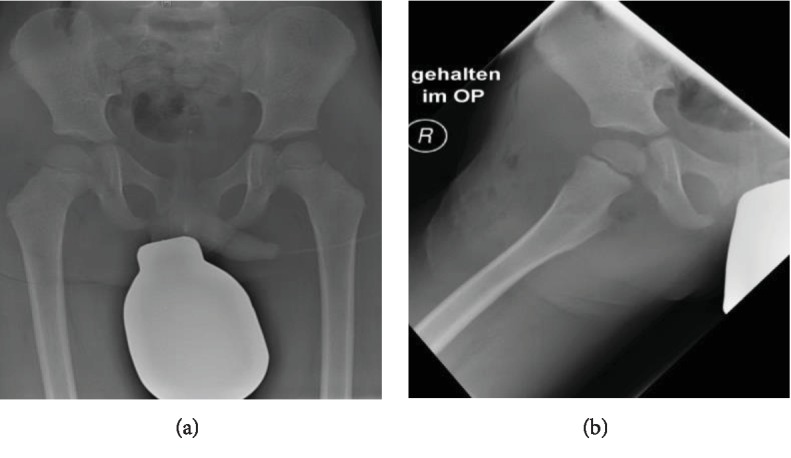
(a, b) Intraoperative X-rays showing the complete reduction in a.p. (a) and axial (b) view.

The postoperative X-rays showed a symmetrical articulation in the hip joints (Figures 5(a) and 5(b)).

The treatment plan started with three days of immobilisation followed by mobilisation with our physiotherapists. In contrast to most of the other cases, pain-adapted full weight bearing was authorized right from the first postoperative day. Even after mobilisation, there were no clinical signs which might indicate a tendency for redislocation. The patient was discharged 4 days after operation. The patient was mobilised with crutches on the ward and suffered only from mild pain levels up to VAS 3/10.

**Figure 6:**
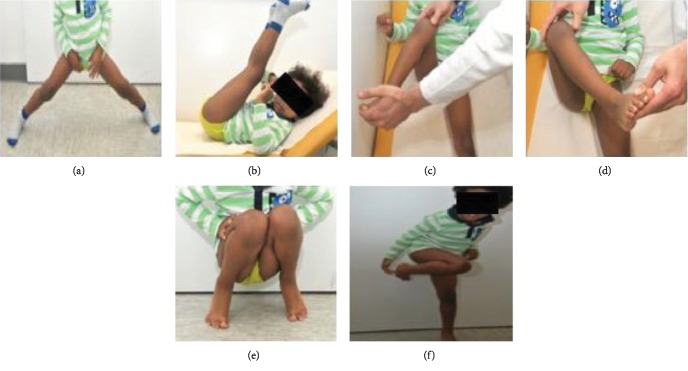
(a–f) Clinical findings especially the range of motion two months after injury.

The patient presented for another MRI scan two months after injury to rule out any signs for femoral head malperfusion, as planned before. The MRI showed normal perfusion without any hints for femoral head necrosis. The physical examination showed a symmetric pain-free range of motion of the right as well as the left hip and knee joint (Figures 6(a)–6(f)). The patient was able to walk with full weight bearing without any complaints. The mother reported that he was acting totally normal during daily activities and while playing with his friends. We instructed the mother to carefully look for signs of femoral head malperfusion.

## 3. Discussion

Traumatic hip dislocations in children are quite rare. Therefore, the number of studies is quite low and most data in the literature is based on case or case series reports. Regarding the pathophysiological mechanisms which cause traumatic hip dislocations in children, it is known that less force is needed than in adult patients. This is mostly due to the higher laxity of the capsular ligament complex. Besides fateful trauma mechanisms, child abuse has to be considered in children with traumatic hip dislocations. The dorsally dislocated hip accounts for about 96% of the cases [5]. It is known that hip dislocations in children often occur along with injuries to the femoral head ligament and avulsion fractures of the femoral head or acetabular fractures; therefore, an additional MRI scan is necessary after reposition [4, 6, 7].

From the current literature, we know that the risk of developing a femoral head necrosis increases with the timeframe between injury and sufficient reposition. Mehlmann and colleagues described a twentyfold higher risk for femoral head necrosis if the reposition is performed with a delay of more than 6 hours after trauma [6, 8]. This emphasizes the need for immediate reduction [8]. In about 90% of the cases, a sufficient closed reduction is possible. There is some evidence that ultrasound-guided reduction may be an option in patients where closed reduction is difficult [9]. If closed reduction is not possible, surgery with open reduction should be performed immediately. The main reasons for insufficient closed reductions are incarceration of osteochondral fragments, the labrum, or the femoral head ligament [10–12]. Therefore, a preoperative multislice radiographic analysis could be helpful [4]. There is different evidence in the literature whether a CT scan or an MRI should be performed. We think that due to the fact that especially in young children (8 years or younger) the informative value of the MRI scan regarding osteochondral lesions or injuries of the capsular complex is higher and radiation protection matters even more than in adults, an MRI scan is the best diagnostic option [11]. This is supported by the data of Kim et al. which showed a diagnostic advantage for the detection of structural injuries for the MRI. And besides the diagnostic advantage, the exposure to radiation should be as low as possible in children [11].

Besides the classic anterior and posterior approach to the hip joint, Morris and colleagues as well as Eberhardt and colleagues described an arthroscopic technique [13, 14]. Due to the low number of cases, none of the studies has enough power to clearly recommend an approach. We prefer the open reduction due to the fact that the overview is much better and the surgeon is able to address possible concomitant injuries in the same operation.

The recommendations for postreduction treatment vary from cast immobilisation for 6 weeks or intermittent extension treatment to early functional treatment with pain-adapted mobilisation [10, 15–17].

One of the main risks after dislocations of the hip joint is the avascular necrosis (AVN) of the femoral head. According to the current literature, the risk is accounted around 5% (up to 17%) and AVN is mainly associated with the timeframe to proper reduction. Even though AVN of the femoral head can occur up to three years after trauma, an MRI scan four to six weeks after trauma is currently recommended to check for AVN. Regarding the scan protocols, Vialle and colleagues described that specific sequences could improve the sensitivity for detecting early signs of AVN [4]. Another option is to perform an early CT scan which goes along with higher exposition to radiation. The main problems of AVN of the femoral head in children under twelve years are severe pain and growth disturbances which can occur years after the hip dislocation.

Another complication is injuries to the sciatic nerve. The incidence of sciatic nerve injuries differs from 5 to 20%. Redislocation is also a main complication. Regarding the known evidence, the rate differs significantly between adults and young patients, where the rate is accounted to be around 6% [1, 18–20].

Another fact which we know from studies in adult patient is the risk for posttraumatic osteoarthritis after dislocations of the hip joint, but due to the quite low number of cases and the lack of sufficient follow-up data, there is no good evidence how often this complication occurs after a hip dislocation in the childhood.

Periarticular ossification and development of a Coxa magna are also described as possible complications [20].

Although many complications are possible, hip dislocations in young children are quite benign injuries when treated immediately, and so in most cases, they heal properly without consequence in the sense of a full restitutio ad integrum.

Besides the level of existing evidence being quite low, there are certain facts which seem to be of some importance in the treatment of traumatic hip dislocation in young children:

- The time between trauma and proper reduction is one of the main factors which has an effect on AVN of the femoral head; therefore, immediate reduction needs to be achieved
- If closed reduction fails, immediate surgery with open reduction should be performed
- If there is any doubt that the closed reduction is not sufficient, an additional MRI scan (or CT) is necessary
- There is no clear evidence for the best postoperative treatment (immobilisation versus early functional mobilisation), so this should be considered from case to case with regard to additional injuries (e.g., osteochondral fractures, ligament rupture, or muscle injuries)
- An MRI scan should be performed immediately after reduction as well as 6 weeks after trauma to asses possible malperfusion

## 4. Summary

Traumatic hip dislocation is very rare in young children. The most common cause of hip dislocation is trauma. In every major trauma such as high velocity accidents hip dislocations occur. As a feature of children hip dislocations also occur in minor trauma due to the laxity of the ligaments. In contrast to hip dislocations in adult patients, hip dislocations in young children are more often missed. The most important fact is the achievement of an early proper reduction. In most of the cases, closed reduction is possible, but if not, immediate open reduction should be performed. Reasons for impossible closed reduction are incarceration of osteochondral fragments, the labrum, or the ligament of the femoral head.

An MRI scan immediately after reduction with regard to additional injuries is advisable as well as an MRI scan around 6 weeks after trauma to check for early signs of complications.

The main complications after traumatic hip dislocation are avascular necrosis of the femoral head, redislocation, and early osteoarthritis.

There is no clear evidence for postreduction treatment so that this should be considered from case to case especially with regard to additional injuries such as osteochondral fractures or injuries of the capsular ligament complex or muscles.

## References

[1] Hamilton P. R., Broughton N. S. (1998). Traumatic hip dislocation in childhood. *Journal of Pediatric Orthopedics*.

[2] Barlebo H., Cristensen J., Stocklund K. E. (1962). Traumatic hip dislocation with fracture of the posterior border of the acetabulum. *Ugeskrift for Laeger*.

[3] Rieger H., Pennig D., Klein W., Grunert J. (1991). Traumatic hip dislocation during the years of growth. A review of the literature. *Zentralblatt für Chirurgie*.

[4] Vialle R., Pannier S., Odent T., Schmit P., Pauthier F., Glorion C. (2004). Imaging of traumatic dislocation of the hip in childhood. *Pediatric Radiology*.

[5] Fernandez F. F., Wirth T., Eberhardt O. (2012). Acute traumatic and especially neglected traumatic hip dislocations are very rare in children. *Unfallchirurg*.

[6] Mehlman C. T., Hubbard G. W., Crawford A. H., Roy D. R., Wall E. J. (2000). Traumatic hip dislocation in Children. *Clinical Orthopaedics and Related Research*.

[7] Struwind C. M., von Ruden C., Thannheimer A., Buhren V., Schneidmueller D. (2018). Relevance of MRI after closed reduction of traumatic hip dislocation in children. *Zeitschrift für Orthopädie und Unfallchirurgie*.

[8] Banskota A. K., Spiegel D. A., Shrestha S., Shrestha O. P., Rajbhandary T. (2007). Open reduction for neglected traumatic hip dislocation in children and adolescents. *Journal of Pediatric Orthopedics*.

[9] Terjesen T. (1992). Closed reduction guided by dynamic ultrasound in late-diagnosed hip dislocation. *Journal of Pediatric Orthopedics*.

[10] Barquet A. (1979). Traumatic hip dislocation in childhood. A report of 26 cases and review of the literature. *Acta Orthopaedica Scandinavica*.

[11] Thanacharoenpanich S., Bixby S., Breen M. A., Kim Y. J. (2020). MRI is better than CT scan for detection of structural pathologies after traumatic posterior hip dislocations in children and adolescents. *Journal of Pediatric Orthopedics*.

[12] Barquet A. (1982). Traumatic anterior dislocation of the hip in childhood. *Injury*.

[13] Eberhardt O., Fernandez F. F., Wirth T. (2012). Arthroscopic reduction of the dislocated hip in infants. *Journal of Bone and Joint Surgery British Volume*.

[14] Morris A. C., Yu J. C., Gilbert S. R. (2017). Arthroscopic treatment of traumatic hip dislocations in children and adolescents: a preliminary study. *Journal of Pediatric Orthopedics*.

[15] Nirmal Kumar J., Hazra S., Yun H. H. (2009). Redislocation after treatment of traumatic dislocation of hip in children: a report of two cases and literature review. *Archives of Orthopaedic and Trauma Surgery*.

[16] Petrie S. G., Harris M. B., Willis R. B. (1996). Traumatic hip dislocation during childhood. A case report and review of the literature. *American Journal of Orthopedics*.

[17] Podesek S., Niedzielski W. (1991). Traumatic hip dislocation in children. *Chirurgia Narzadów Ruchu i Ortopedia Polska*.

[18] Barquet A. (1982). A vascular necrosis following traumatic hip dislocation in childhood: factors of influence. *Acta Orthopaedica Scandinavica*.

[19] Hughes M. J., D’Agostino J. (1996). Posterior hip dislocation in a five-year-old boy: a case report, review of the literature, and current recommendations. *The Journal of Emergency Medicine*.

[20] Meena S., Kishanpuria T., Gangari S. K., Sharma P. (2012). Traumatic posterior hip dislocation in a 16-month-old child: a case report and review of literature. *Chinese Journal of Traumatology*.

